# MASSAG model: Towards an integrative neuroscience framework linking emotional trauma, pain, and mechanisms of force-based manipulations

**DOI:** 10.1016/j.neubiorev.2025.106517

**Published:** 2025-12-09

**Authors:** Alex Jinich-Diamant, Benedetta Albinni, Joel N. Fishbein, Eric Jacobson, Victoria E. Abraira, Suzi Hong, Austin C. Korgan, Anna-Maria Mazzieri, Laura Case

**Affiliations:** a Department of Anesthesiology, UC San Diego, United States; b Department of Cognitive Science, UC San Diego, United States; c Center of Excellence for Stress and Mental Health, Department of Veteran Affairs, United States; d VA San Diego Healthcare System, United States; e Department of Psychiatry, UC San Diego, United States; f Global Health and Social Medicine, Harvard Medical School, United States; g Neuropeptide Laboratory, Department of Gastroenterology, Beth Israel Lahey, United States; h Department of Cell Biology and Neuroscience, Rutgers The State University of New Jersey, United States; i Herbert Wertheim School of Public Health & Human Longevity Science, UC San Diego, United States; j Department of Psychiatry, UC San Diego, United States; k Department of Psychiatry, University of Colorado Anschutz Medical Campus, Aurora, CO, United States; l The ST School, Exmouth, Devon, UK; m Centre for PAIn (Pain and Active Inference), Research Health Sciences University, Bournemouth, UK

**Keywords:** Force-based manipulation, Manual therapies, Somatosensory, Trauma, Stress

## Abstract

The frequent comorbidity of chronic pain, affective disorders, and trauma histories suggests shared mechanisms, and opportunities for interventions that target their overlap. Force-based manipulations (FBMs) of the soft tissues such as massage and fascial manipulation are especially relevant given their dual impact on sensory and affective mechanisms. This paper synthesizes current evidence on the distributed somatosensory effects of emotional trauma, evaluating how trauma and stress reshape neural, immune, and connective tissue functions, altering sensory perception and pain processing. By elucidating known as well as plausible mechanisms, we aim to provide a foundation for advancing research on how FBMs of the soft tissues may counter stress and trauma-related alterations in the somatosensory system. We then propose the MASSAG (Mechanisms of Affective Somatosensory Soothing for Allostatic Gain) model, which is intended to provide a framework for understanding the therapeutic benefit of manual therapies and to guide future research in this field. This integrative framework conceptualizes how manipulation of the soft tissues engage both sensory-afferent and cognitive-affective pathways ideally situated to reshape predictive models of somatosensory experience and counter the long-term effects of trauma and pain.

## 1. Introduction

Emotional trauma exerts body-wide effects that alter the experience of pain and touch in ways that can persist over very long terms. For example, post-traumatic stress disorder (PTSD) can alter the processing of sensory stimuli, causing hyper- or hypo-responsivity that may contribute to hyperarousal and dissociation (Fleming et al., 2024). Indeed, adverse childhood experiences (ACEs) double the risk of chronic pain in childhood, adolescence, (Groenewald et al., 2020) and adulthood (Dalechek et al., 2024). Trauma history renders pain more unpleasant (Gómez-Pérez and López-Martínez, 2013) and reduces the experience of pleasant touch, (Devine et al., 2020; Strauss et al., 2019) and among those with chronic pain, psychological trauma is associated with hyperalgesia (Tesarz et al., 2015). Amplified threat detection mechanistically links pain and trauma, (Elman and Borsook, 2018; Burke et al., 2017; Strigo and Simmons, 2024; Pinto et al., 2023a; Korgan et al., 2025) while psychological treatments demonstrate the powerful role of emotional processes in pain perception (Urits et al., 2019; Ashar et al., 2022).

Most standard-of-care clinical approaches to comorbid trauma-related symptoms and chronic pain involve psychological therapies and/or medication. Here, we propose that touch-based therapies are underappreciated but ideally positioned complementary approaches to intervene on the effects of trauma on the body and the somatosensory system. Touch and force-based therapies like massage, structural integration, and myofascial therapy are widely sought (U.S. lifetime use ~13 %) (Sundberg et al., 2017) and can improve both pain and stress, (Sundberg et al., 2017; Crawford et al., 2016) including postsurgical pain, (Miozzo et al., 2016) fibromyalgia pain, (Kundakci et al., 2022) and short-term musculoskeletal pain relief, (Bervoets et al., 2015; Furlan et al., 2015) yet biomedicine has largely neglected these approaches. Despite this, interest in their health effects has risen steeply following major advances in the molecular and neural underpinnings of touch, (Löken et al., 2009; Morrison et al., 2010; Sharma et al., 2020; Meltzer et al., 2021; Fede et al., 2022) public interest in nonpharmacologic approaches to pain management, and research highlighting the negative health effects of social isolation touch deprivation (Croy, 2021; Von Mohr et al., 2021).

A variety of force-based manipulations (FBMs) target the soft tissues. Some of these have substantial research literatures, including massage, (Mak et al., 2024) myofascial release, (Lv and Yin, 2024) trigger point, (Müggenborg et al., 2023) manual lymphatic drainage, (Palmer, 2024) strain-counter-strain, (Wong et al., 2014) reflexology, (Embong et al., 2015) and structural integration (Jacobson, 2011; Stall and Teixeira, 2014; Jacobson et al., 2015; Jędrzejewski et al., 2020). Soft tissue manipulations like acupressure, (Li et al., 2024) shiatsu, (Robinson et al., 2011) and tuina (Xu et al., 2024) are associated with traditional Asian medicine. Others, like deep friction, (Joseph et al., 2012) manual traction, cross friction, dermal-fascial restoration, (Mettler, 1994) and dermal-neuro modulation, (Jacobs, 2025) have minimal or no peer-reviewed literature.

Soft tissue FBMs differ from other mind-body practices like yoga or meditation by the application of touch or force to the body surface, modulating the somatosensory system through bottom-up (sensory-afferent) and top-down (cognitive-affective) mechanisms. This dual approach is particularly well-suited for persistent pain and trauma-related conditions, which frequently involve both peripheral and central mechanisms. To understand how these bidirectional impacts may improve pain and trauma-related symptoms, we must first consider how these experiences alter the somatosensory system.

We first review the effects of trauma on the somatosensory, immune, and fascial systems and discuss implications for touch and pain perception. Next we explain how trauma can alter the anticipatory mechanisms of the nervous system over long terms. Finally, we propose the ‘MASSAG’ model, an integrative theory informed by predictive coding for how soft tissue FBMs may recalibrate the nervous system to alleviate trauma-related symptoms. By elucidating potential mechanisms of soft tissue FBMs, we aim to inspire mechanistic and clinical research investigating the complex interplay between brain, body, and psychosocial context.

## 2. Effects of emotional trauma and FBMs on the somatosensory system

In this section, we consider evidence that emotional trauma may reorient the somatosensory system on a long-term basis towards the anticipation of threat and heightened nociceptive reactivity. We review the effects of emotional trauma on the hypothalamic-pituitary adrenal (HPA) axis, the immune fascial, and peripheral and central nervous systems, and their potential contributions to persistent pain.

### 2.1. Nervous and Immune Systems

We begin with a brief overview of nervous and immune system interactions while focusing on the HPA axis, and consider the impacts of trauma and the modulatory effects of soft tissue FBMs. The autonomic nervous (ANS) system maintains homeostasis through a bi-phasic activation of the sympathetic and parasympathetic nervous systems. Real, perceived, or anticipated threats activate the sympathetic nervous system (SNS) to release epinephrine, increasing blood pressure and muscle blood supply and suppressing gastrointestinal activity (Oubaid, 2023). SNS activation also shifts the immune system into a heightened pro-inflammatory state as a protective mechanism against potential physical injury (Russell and Lightman, 2019). Meanwhile along the HPA axis, the release of hypothalamic corticotropin-releasing factor (CRF) stimulates pituitary secretion of adrenocorticotrophic hormone (ACTH) and leads the adrenal gland to release cortisol to systemically increase glucose availability and utilization, and to modulate inflammation (Chu et al., 2024). CRF and ACTH release are under negative feedback control through the central monitoring of cortisol levels. When the perception of threat pas, parasympathetic activation restores homeostasis (Chu et al., 2024). Importantly, this coordinated recovery and the associated extinction of contextual fear (Gilmartin et al., 2014) depend on a normally functioning HPA axis, on adequate neuroplasticity, on normal communication between the prefrontal cortex (PFC), amygdala, and hippocampus, and on physiologic rates of clearance of pro-inflammatory and nociceptive amplifiers from the interstitial matrices of soft tissues.

Physiologically normal changes in the ANS and HPA axis function are key to *allostasis,* the body’s ongoing maintenance of homeostasis by adjusting physiological and behavioral activations in response to actual and predicted stressors. Repeated or prolonged responses to stressors increases *allostatic load*, the cumulative physiological consequences of attempts to adjust and adapt to stress. Suffering trauma beyond a certain magnitude and/or frequency imposes significant allostatic load and can alter ANS and HPA axis functions longer-term. The brain’s predictive threat appraisal systems can be altered, causing sympathetic activation to be disproportionately triggered by non-threatening stimuli (Azevedo et al., 2024; VanItallie, 2002) (see Section 3). Higher life trauma is associated with lower basal cortisol levels, an effect mediated by increased acute stress responses in the amygdala and hippocampus (Seo et al., 2019). Trauma during infancy delays cortisol recovery from stress, while trauma in later life alters circadian cortisol regulation (Kuhlman et al., 2015). However associations between PTSD and HPA axis dysregulation are mixed, and further investigation is warranted (Speer et al., 2019).

A dysregulated HPA axis impacts immune and inflammatory regulation, which can in turn amplify pain via nociceptive sensitization. Prolonged immune activation is driven by continuous production of pro-inflammatory cytokines like IL-6 and TNF, which produce persistent inflammation, leading to tissue damage, and dysfunction in various organs and systems each of which can in turn drive chronic pain (Chu et al., 2024; Chen et al., 2017; Fink and Singer, 2017; Hannibal and Bishop, 2014). Sustained inflammation-driven sensitization increases the excitability of sensory neurons, causing neuronal hyperactivity, hyperalgesia, and chronic pain (Reichling and Levine, 2009).

Inflammation-induced mitochondrial disturbances enhance nociceptive signaling and contribute to hyperalgesic priming of nociceptive afferents (Kandasamy and Price, 2015). Inflammation also upregulates mechanosensitive ion channels such as Piezo1, Piezo2, and TRPV4, which are densely expressed in sensory mechanoreceptors of the skin and deeper fascia, and in dorsal root ganglion neurons, enhancing the sensitivity by decreasing stimulation thresholds and inducing plasticity in translational control pathways (Mikesell et al., 13 2023; Murthy et al., 2018; Szczot et al., 2018; Zhao et al., 29 2015; Solis et al., 2019; Scheraga et al., 1 2016; Mikesell et al., 2024). Chronic inflammation of peripheral tissues initiates and maintains central sensitization in all these ways.

Repeated environmental stressors can cause a lasting elevation of circulating proinflammatory cytokines (Maes et al., 1998) and induce a persistent hyperalgesic state (Iwai-Liao and Senba, 2006). This sustained and excessive inflammatory response is observed in PTSD, (Sun et al., 2021) a disorder that is also associated with reduced anti-inflammatory factors and shares gene expression commonalities with immune disease (Sun et al., 2021). Recent studies link neuroimmune activation to chronic pain (Loggia, 2024) and neuroinflammatory action to fibromyalgia (Mueller et al., 2022)and to negative affect in chronic pain mediated by, glial activation (Albrecht et al., 2021). These effects may differ in the short versus long-term. Additional research is needed to understand how emotional trauma impacts the HPA axis and immune system over different time periods.

With regard to therapies, massage has been linked to lower pain and inflammation following physical trauma in rodents in both repetitive injury models and post-surgery Chapelle et al., (2013); Bove et al., (2016) and following acute musculoskeletal pain from sports-related injuries (Crane et al., 2012; Waters-Banker et al., 2014; White et al., 2020). Some studies have also observed reductions in inflammatory markers and pathways such as sVCAM-1 (Wang et al., 2023) and TLR4 (Liu et al., 2022). However, we lack studies of massage’s effects on emotional trauma.

In clinical and research contexts, patients frequently describe FBMs, particularly massage, as profoundly calming and inducing deep relaxation and stress alleviation (Moraska et al., 2010; Meier et al., 2020). Studies have associated massage to transient decreases in salivary cortisol compared to no-treatment controls, even after a single session, (Lee et al., 2024) which suggests parasympathetic activation (Diego and Field, 2009) and a temporary downregulation of HPA axis activity (Moraska et al., 2010; Golden et al., 2011; Rapaport et al., 2010). This transient cortisol reduction supports the role of massage in acute stress recovery, supporting allostasis and preserving the integrity of future stress responses (Rapaport et al., 2012).

Soft tissue FBMs have also been proposed to regulate the ANS via oxytocin (OT) release (Uvnäs-Moberg et al., 2004; Cerritelli et al., 2020; Van Puyvelde et al., 2019). OT can directly modulate the immune system, (McParlin et al., 2022) other hypothalamic–pituitary–immune axes, and the ANS (Li et al., 2017). OT is also involved in social touch pathways that promote social interaction (Yu et al., 2022). Notably, OT levels are often lower in individuals with a history of trauma (Donadon et al., 2018a; Heim et al., 2009). This suggests that interventions that increase OT levels might counter HPA and immune system dysregulation and improve social functioning.

Although the long-term effects of soft tissue FBMs on HPA axis activity are not clear, a randomized controlled trial found that repeated exposure to massage therapy in healthy individuals lowered vasopressin and cortisol and increased OT levels, (Rapaport et al., 2012) suggesting a dose-response relationship in HPA modulation. Less frequent sessions showed shifts in lymphocyte markers and cytokine profiles suggestive of a separate immunomodulatory pathway.

### 2.2. Muscle tissue and associated sensory afferents

Effects of trauma extend beyond the nervous system and neuroendocrine system to the peripheral organs and tissues, causing both short and long-term changes. Sympathetic activation leads to a rapid and pronounced release of epinephrine (EPI) and norepinephrine from the adrenal medulla, (Azevedo et al., 2024; VanItallie, 2002) followed almost immediately by norepinephrine release from postganglionic sympathetic neurons innervating various tissues, including smooth muscle (Burnstock, 2008). Activated adrenergic receptors initiate intracellular cAMP signaling cascade that increases muscle contractility, a key component of the “fight or flight” response (Chu et al., 2024). Sympathetic activation can also enhance TGF-beta1 expression, promoting myofibroblast contractility (Bhowmick et al., 2009). Trauma of great magnitude and/or frequency may initiate “sympathetic overshooting” in which the body is flooded with large quantities of EPI and NOR, thus triggering all of these effects with much greater intensity.

Both mental and physical stressors have been shown to elevate physiological stress markers, muscle contractility, and muscle activity as measured by electromyography (EMG) (Lundberg et al., 1994). One prominent example is the masticatory musculature. Stress often increases chewing frequency or involuntary teeth clenching, both behaviors associated with anxiety and psychological distress (Anna et al., 2015) that are linked to temporomandibular disorders, (Ahuja et al., 2018; Zieliński et al., 2018) stress-related tension headaches, (Fumal and Schoenen, 2008) and chronic shoulder and neck pain (Chu et al., 2024; Lundberg et al., 1999). Over time, stress and sustained muscle tension may contribute to muscle and joint degradation, increasing the risk of musculoskeletal injuries, (Allen et al., 2010; Finestone et al., 2008) and may lead to long term alterations in muscle due to chronic contractility and frequent exposure to sympathetic endocrine cascades. In addition, trauma may reduce local perfusion and oxygenation of muscle tissues. Women with PTSD demonstrate greater muscle fatigability and blunted peripheral extraction of oxygen (D’Souza et al., 2024). Similarly, individuals with temporomandibular disorder exhibit elevated stress and reduced masseter oxyhemoglobin values (Puel et al., 2023).

Therapeutically, soft tissue FBMs can exert acute effects on muscle contraction. For example, cervical thoracic manipulation promotes muscle relaxation in patients with chronic neck pain (Bakar et al., 2014) and massage of the medial gastrocnemius temporarily reduces muscle stiffness (Eriksson Crommert et al., 2015). Similarly, myofascial release has been shown to decrease thoracolumnar fascia thickness (Devantéry et al., 2023). Most observed reductions in muscle tension or fatigue are short-term, and robust evidence for persistent changes remains limited. In rats, however, massage remodels tissue by increasing collagen fibrils in the tendons (Kassolik et al., 2013) and by reducing visceral adhesions after abdominal surgery (Bove and Chapelle, 2012; Bove et al., 2017). Moreover, following hypertrophic stimulation, myogenic progenitor cells within the extracellular matrix (ECM) in adult skeletal muscle release extracellular vesicles (EVs) with microRNAs that promote maintenance and adaptation of the local ECM (Fry et al., 5 2017; Van Pelt et al., 1 2020). While these findings cannot be extrapolated to humans, they raise the possibility that massage influences muscle physiology through myofascial remodeling.

FBMs also modulate circulation and tissue oxygenation, with consequences for muscle function and pain. In humans, techniques such as effleurage (a light stroking technique) acutely increase skin microcirculation/perfusion and modify cardiac, respiratory, and myogenic factors that support microcirculatory homeostasis (Monteiro Rodrigues et al., 2020). Similarly, rolling massage improves skeletal muscle oxygenation and parameters associated with microvascular reactivity (Soares et al., 2020). This is significant since muscle hypoxia activates muscle nociceptors (Mense and Stahnke, 1983) and may result in pain and muscle tension. Additionally, oxidants (induced by hypoxia) increase the excitability of sensory neurons during inflammation, (Takahashi and Mori, 2011) further contributing to muscle discomfort and contraction. This is especially relevant in chronic pain conditions like fibromyalgia (FM) where muscle oxygenation is impaired (Lund et al., 1986; Vierck, 2006; Bagis et al., 2005) and may alter pain (Yildiz et al., 2004; Efrati et al., 2015).

Some effects of FBMs may also be mediated by deeper tissue afferents detecting warmth and pressure, which have been proposed to convey feelings of physical and social safety. Feelings of safety may derive from mammalian affiliative behaviors like huddling and snuggling, which promote thermoregulation and reduce separation distress (Morrison, 2016a). Warmth and deep pressure are typically experienced as pleasant (Case et al., 2020; Rolls et al., 2008) and can inhibit the fear response and serve as a safety signal (Case et al., 2020; Hornstein et al., 2022; Baumgartner et al., 2023; Morrison, 2016b) that can reduce sympathetic arousal, (Reynolds et al., 2015) increase parasympathetic activity, (Diego and Field, 2009) decrease pain, (Honigman et al., 2016) and improve sleep (Yu et al., 2024). Deep pressure targeting muscle tissue is often preferred in therapeutic contexts by individuals with Fibromyalgia (clinical observation, author A.M.) and may regulate the nervous system-possibly since deep pressure is not *Piezo2* mediated (Case et al., 2021) and as such may not be impacted by differences in early life sensory input that have been shown to alter the development of sensory afferents for gentle touch in rodent models (Santiago et al., 2023).

### 2.3. The fascial system and associated sensory afferents

Fascial tissues are now understood to constitute a body-wide, three-dimensional visco-elastic system across which biomechanical forces, fluids, and a variety of cell types communicate and travel. Its primary components are a matrix of highly hydrophilic hyaluronic gel, embedded collagenous and elastic fibers, and a variety of cell types of which fibroblasts, and sensory and autonomic neurons are the most numerous (Stecco, 2014; Pirri et al., 2022).

Physical trauma, inflammation, and biomechanical strain alter the viscosity of the fascial gelatin, the elasticity of fibers, and the motility and secretions of fibroblasts. They can also cause densification, in which the hydration of the gelatinous component is reduced to the point that fluid migration through the fascia - a major feeder of lymphatic drainage - is almost halted (Pratt, 2021; Hughes et al., 2019; Langevin, 2021). In response to injury or stress, fibroblasts can transform into myofibroblasts capable of mobility, and extension of pseudopodia which can readily adhere to the underlying gelatin, and exert long-term tensional forces across fascial sheets. In that way fascial sheets can have an active, varying tonus. Increases in the proportion of inelastic to elastic fibers can also contribute to densification, and can impede glide between adjacent fascial planes, contributing to nociceptive signaling (Langevin, 2021; Pavan et al., 2014). Trauma-induced remodeling of the fascial matrix via each of these mechanisms can alter the local environment of the sensory and autonomic neurons that richly innervate both superficial (sub-cutaneous) and deep fascia, (Stecco et al., 2019; Fede et al., 2021) potentially contributing to chronic soft tissue pain (Stecco et al., 2013).

FBM practitioners often describe thickening, stiffening and reduced glide (lateral shear strain) of both superficial and deep fascia in chronic pain patients. Multiple studies have confirmed modifications in thoracolumbar fascia of chronic low back pain, (Langevin et al., 2011; Vita et al., 2025; Tamartash et al., 2023; Tomita et al., 2025) with one systematic review showing that higher stiffness, nociceptive innervation, and inflammation are mediated by increased deposition of inelastic collagenous fiber, altered myofibroblast activity, and increased levels of matrix metalloprotease (MMP), proinflammatory cytokines, and immune phenotypes in the hyaluronic matrix (Kondrup et al., 2022).

Some FBM practitioners propose that traumatic memory is ‘stored’ in the fascial system, (Tozzi, 2014) and reviews have hypothesized that, following trauma, released inflammatory mediators may become entrapped in the interstitial, pre-lymphatic pathways or in initial lymphatic vessels. This is plausible because pro-inflammatory cytokines can disable local lymphatic pump mechanisms, impair vascular perfusion via sympathetic activation, and, through TGF-beta 1 expression, contribute to fascial compression of pre-lymphatic pathways (Tuckey et al., 2021). Following physical injury, fascial fibroblasts, myofibroblasts, adipocytes, mast cells, lymphocytes, and vascular cells, all release pro-inflammatory cytokines like IL-1, IL-6, and TNF-alpha (Barsotti et al., 2020). Cytokines then increase the production of MMP, proteases, and reactive oxygen species, (McParlin et al., 2022) which contribute to further inflammation and tissue remodeling (Barsotti et al., 2020). Mechano-receptors, which densely populate both superficial fascia (SF) and deep fascia (DF), may begin to function as nociceptors in response to heightened concentrations of pro-inflammatory factors, contributing to localized soft tissue pain and long term sensitization (Kondrup et al., 2022; Suarez-Rodriguez et al., 2022). Chronic or traumatic stress might induce similar long-term changes via these pathways and similarly contribute to peripheral sensitization.

Several hypotheses for how fascial inflammation may become chronic have been advanced. Sympathetic overshooting could cause fibroblasts to increase their secretion of inelastic fibers with subsequent fibrosis and densification of the fluid matrix, which would reduce or even halt the clearance of pro-inflammatory factors. At the same time released antibodies could bind with myofascial-derived antigen to promote hyperexcitability in the dorsal root ganglia which would contribute to central sensitization (Stecco et al., 2013). Sympathetic hyperactivity caused by emotional trauma may impair the resolution of inflammation, promoting autoimmunity and excessive autoantibody production, which would lead to neuronal hyperexcitability, the activation of satellite glial cells and spinal microglia, and central sensitization (Liptan, 2023).

Recent research additionally suggests that persistent nociceptive activity from visceral or somatic tissues may induce central sensitization in the corresponding spinal segments, leading to reciprocal downstream dysfunction in the myofascial unit (referring to muscle tissue, its surrounding fascia, and associated nerves, blood vessels, and lymphatics) (Sikdar et al., 2023). Central sensitization can activate primary afferents and dorsal root reflexes leading to antidromic release of proinflammatory neuropeptides into the innervated tissues causing local vasodilation, interstitial inflammatory stasis, and impaired fascial gliding. This theory explains common clinical observations of myofascia that is thickened, dehydrated, and lacking normal glide in myofascial pain syndrome (MPS) and similar conditions such as non-specific lower back pain, including deep and diffuse pain, regional tenderness, and comorbidities with visceral, somatic, and psychosocial pathologies (Sikdar et al., 2023). In addition to these peripheral mechanisms, sensitization of the spinal cord can also be modulated and possibly initiated through descending mechanisms, providing a plausible link between stress and emotional trauma and tissue abnormalities.

Therapeutically, myofascial release-techniques can effectively reduce soft tissue pain, (Pawlukiewicz et al., 2022; Ajimsha et al., 2015; Overmann et al., 2024; Casato et al., 2019) and myofascial release therapy improves ultrasound measures of sliding fascial mobility (Tozzi et al., 2011) and decreases the thickness of thoracolumbar fascia (Devantéry et al., 2023). However, the efficacy of soft tissue FBMs in reversing tissue-level and central effects driven by emotional trauma is unclear and presents a significant research gap and opportunity. Clearly, manual interventions can affect fascial structure and function, improve fluid drainage, alleviate chronic inflammation, and modulate nociceptive thresholds. These effects might plausibly contribute to reductions in peripheral sensitization by decreasing the mechanical and biochemical stimuli that sustain nociceptor activation. In addition, mechanical stimulation of fascial receptors might play a role in neuroplastic adaptations that could counteract maladaptive central sensitization caused in part or full by emotional trauma. We emphasize that further research is needed to elucidate the precise neurophysiological and biochemical responses of fascia to FBMs, particularly in the context of emotional trauma and central sensitization.

The foregoing findings and hypothesis suggest that FBMs could alter the influence of the soft tissue matrix on the sensitization of the embedded mechanoreceptors. Traumatic memories must include patterns of sensorimotor stimulation at some level, given that sensorimotor activity is integral to memory traces (Dijkstra and Post, 2015; Barsalou, 1999; Ianì, 2019). While CNS mechanisms of memory are clearly significant, a peripheral, interoceptive component is plausible, and would explain certain clinical phenomena. FBM practitioners often report that clients experience reactivations of traumatic memories during treatment, and that these are often associated with beneficial therapeutic outcomes (author E.J.). Given that such episodes are triggered by manipulation of the fascia, it is plausible that they arise from the effect of those manipulations on a trauma-induced modification of the fascia that is connected to such memories. We propose that patterns of mechanosensory simulation that attended traumatic episodes may somehow be encoded in an altered relationship between those neurons and the surrounding fascial matrix. Given what is known about the effects of inflammation and sympathetic flooding, it is plausible that traumatic episodes might produce extreme, long-term densification and fibrosis of the matrix surrounding arrays of mechanoreceptors that were stimulated by a traumatic episode. On that hypothesis, restoring elasticity and the fluid clearance of cytokines and nociceptive amplifiers might allow those mechanoreceptors and the spinal horns which they feed to be de-sensitized, and regain the capacity to respond to current stimulations, rather than past traumatic memories. That would reduce thresholds for the reactivation of traumatic memories and for predictions of threat and pain. New research methods would be essential to investigate the relationship between alterations of the fascial matrix subsequent to sympathetic flooding, and possible alterations in the embedded mechanoreceptors. Mechanistic research and clinical trials will also be needed to determine the extent to which soft tissue FBMs can reverse tissue-level and central effects of emotional trauma, alone or in tandem with psychological therapies.

### 2.4. Skin and associated sensory afferents

The skin, the outermost and largest sensory organ in the human body, serves as a vast transduction zone for somatosensory stimuli. This includes C-fiber nociceptors but also C-tactile (CT) fibers, a subset of unmyelinated sensory afferents that respond to slow, gentle stroking at skin temperature (Morrison et al., 2010; Vallbo et al., 1999; Gossrau et al. 2021; Nees et al. 2019; Case et al. 2016). CT stimulation is generally pleasant, (Löken et al., 2009) induces positive affect, (Pawling et al., 2017) and can reduce pain, (Liljencrantz et al., 2017) leading to its characterization as a form of ‘affective touch’.

Besides sensitizing peripheral tissues and the sensory neurons innervating them (Section 2.1), trauma can alter the development and sensitivity of somatosensory terminals in the skin. The deletion of *Piezo2*– the mechanosensitive ion channel underlying most light touch sensation–causes major changes in end organ structure and central targeting of somatosensory afferents, (Santiago et al., 2023) with the greatest alterations in CT fibers. The striking implication is that touch input plays a key role in the transcriptional maturation of somatosensory neurons, especially on afferents conveying social touch. Early life trauma, which is often associated with reductions in soothing touch and increases in nociceptive touch (such as through neglect, physical violence, and sexual abuse), may therefore alter peripheral somatosensory development and confer a predisposition to pain perception. Indeed, a study of neonatal mouse pups found that early life stress altered transcriptional and electrophysiological signatures of immature dorsal root ganglia cells and led to touch and pain hypersensitivity (Harbour et al., 2025).

Trauma experienced specifically during adolescence can lead to unique programming of the skin. Inflammatory processes are often driven by cell-free mitochondrial DNA (cf-mtDNA), a byproduct of mitochondrial dysfunction that acts as a danger signal (Nidadavolu et al., 23 2023). During cellular stress or damage, cf-mtDNA can be released into the bloodstream, triggering the innate immune response and contributing to conditions such as major depressive disorder, suicidality, autoimmune diseases, and chronic pain (Reichling and Levine, 2009; Kandasamy and Price, 2015; Willemen et al., 31 2023). Trauma occurring during specific developmental windows has been associated with significant alterations in EVs, circulating cf-mtDNA levels, (Morrison et al., 1 2022) and altered expression of keratinocyte and Merkel cell-related genes from the 17q21 gene cluster in association with traumatic sexual violence during adolescence (Morrison et al., 1 2022; Korgan et al., 2024). The consequences of this tissue reprogramming are not yet clear. All of these mechanisms occur in and interact with changes in the superficial, sub-dermal fascia which is the immediate environment of receptors and axons.

With respect to possible therapeutic mechanisms, the effects of FBMs on EV expression are largely unknown and present an interesting research opportunity. FBMs directly activate mechanoreceptors and nerve endings in skin cells such as Merkel cells, keratinocytes, and melanocytes, (Zimmerman et al., 2014; Walsh et al., 2015) in components of the myofascial unit, immune cells, macrophages and neutrophils, and in the dorsal root ganglia (DRG). All these cellular stimulations can alter the expression of EVs. As discussed earlier, in animal models, hypertrophic stimulation can lead myogenic progenitor cells to release EVs containing microRNAs that help remodel and adapt the myofascial matrix (Fry et al., 5 2017; Van Pelt et al., 1 2020). Similar intercellular communications might exist within the skin and myofascial unit to promote remodeling in response to touch and pressure, facilitating tissue adaptation and repair.

In addition, touch may be capable of inducing structural plasticity in the somatosensory system. Recently, a study imaging genetically-labelled sensory fibers in mice demonstrated an ingrowth of nociceptive afferents after nerve injury (Gangadharan et al., 2022). Denervated skin lost sensation, gradually recovered sensitivity, and then developed allodynia and aversion to gentle touch several months later. Low-threshold touch afferents did not reinnervate, leading to abnormal innervation of tactile end organs by nociceptors alone. This demonstrates the relevance of structural plasticity in chronic pain phenotypes and raises questions about whether either trauma or touch and FBMs could influence this sort of structural plasticity. It will be important to study whether non-painful touch input might alter the dominant growth of nociceptors after injury, and the effects of specific forms of affective touch such as CT-targeted touch. Furthermore, it will important to study these processes across the lifespan; some sensory systems exhibit critical stages for tuning of activity-dependent gene expression in response to environmental cues (Bell et al., 2014; Tsukahara et al., 2021; Harris et al., 2023).

FBM-based touch, such as rubbing, squeezing, and pressure, may also alleviate pain through neural effects. Touch-induced pain modulation is most often explained by gate control theory, wherein A-fiber input gates ascending nociceptive projections (Melzack and Wall, 1967; Field et al., 2007). FBMs also typically activate CT fibers, which are associated with positive affect and pain reduction (Liljencrantz and Olausson, 2014; Meijer et al., 2022). CT input also activates several brain regions involved in pain interpretation and descending pain modulation (summarized in (Meijer et al., 2022)). In rats trained on repetitive tasks to induce injury, massage prevents pain-related behaviors and reduces spontaneous activity in nociceptive C-fibers (Bove et al., 2019).

Whether massage or other soft tissue FBMs can alter the longer-term development or function of somatosensory afferents, including CT fibers, is an open question. The impact of sensory input on sensory development of affective touch reviewed above may partly explain why affective touch in infancy increases attachment and reduces the future risk of psychopathology (Norholt, 2020). Just as inflammation can alter sensory afferent function, pleasant CT touch may alter somatosensory gene expression or spinal cord processing of touch valence, such as via reductions in inflammation or release of oxytocin. Whether extended touch provided through soft tissue FBMs could complete somatosensory maturation processes left incomplete during development- or shift the responsiveness of tactile afferents to reduce nociceptive activation and the consequent risk of chronic pain-constitutes a significant research gap and opportunity.

If affective touch plays a role in programming the sensory system during development, the activation of CT fibers during massage might extend beyond local, immediate effects such as reducing inflammation or improving muscle function. Beyond simply contributing to pain relief, CT fibers might facilitate a cognitive reappraisal process whereby sensory experiences, initially perceived as painful or tense, are reinterpreted and assigned a positive emotional significance such as comfort or safety, contributing to long term reorganization of the sensory system and enhancing the brain’s ability to modulate pain perception and to integrate sensory information.

### 2.5. Brain and cognitive effects

Persistent stress and neuroinflammation alter brain-wide connectivity and structure, (Kaul et al., 2021) and in particular impair brain regions involved in executive function and stress response (McEwen and Gianaros, 2010; Wang et al., 2019). Elevated cortisol levels damage the hippocampus and disrupt neuroplasticity and memory, (McEwen et al., 2016) while stress impairs emotional regulation and cognitive control via the prefrontal cortex (PFC) (McEwen et al., 2016). Changes in the amydgala increase threat detection and fear learning, sustaining hyperarousal (Kaul et al., 2021). Stressful and traumatic events experienced during childhood are linked to chronic multisite pain and reduced hippocampal volume, (Lobo et al., 2022) with higher severity correlating to increased pain intensity. Neuroimaging confirms volume loss in the hippocampus, PFC, and amygdala in individuals diagnosed with PTSD (Henigsberg et al., 2019). Loss of hippocampal volume is also highly correlated with major depression, (Videbech and Ravnkilde, 2004) emotional trauma is associated with gray matter loss in the anterior cingulate, hippocampus, and parahippocampal gyrus (Papagni et al., 2011). Stress-related brain changes show stronger effects in females (McManus et al., 2022).

Threat reactivity pathways and pain are closely associated, (Pinto et al., 2023a) and emotional trauma alters the experience of touch and pain. PTSD has been linked to altered processing of neutral touch as either threatening or painful (Badura-Brack et al., 2015). In adolescent mice, early life stress from maternal separation increases pain sensitivity. This effect is linked to altered neural activity in the anterior insular cortex and its communication with anterior cingulate cortex; silencing specific interneurons in this circuit reduces hyperalgesia (Li et al., 2025). Individuals with PTSD are predisposed to chronic pain, (Defrin et al., 2008; Moeller-Bertram et al., 2014) and reductions in touch pleasantness are observed in both chronic pain patients (Morrison et al., (2010); Vallbo et al., (1999); and trauma exposed individuals (Devine et al., 2020; Strauss et al., 2019). These observations are consistent with a recent theory of the nociplastic pain condition FM (fibromyalgia) that proposes that an overactive “threat system” and underactive “soothing system” sustain hypervigilance in FM patients, amplifying both pain and negative emotional states (Pinto et al., 2023a). Overactive threat prediction may amplify expectations for pain and reduce predictions of pain relief (Strigo and Simmons, 2024) (see Section 3).

The evidence for therapeutic effect of FBMs on trauma-related cognitive changes is limited (Villemure et al., 21 2013). However, we can identify two ways in which soft tissue FBMs might plausibly reverse some of the long-term effects of trauma. The first of these is through safe exposure to touch. Exposure therapy is a well-established and effective approach for treating anxiety disorders and PTSD via controlled, progressive exposure to feared stimuli (Craske et al., 2022; Abramowitz et al., 2019). From this perspective, FBMs may gradually reduce threat reactivity by promoting the experience of touch, even very firm touch, as safe. Indeed, touch can regulate stress and recovery from fear, (Kearney and Lanius, 2022) and one study that integrated romantic partner touch with CBT for PTSD successfully reduced threat responses to (Baggett et al., 2017). Even though soft tissue FBMs do use non-intimate touch, trauma-informed FBM interventions still require specialized clinical training to ensure safety and prevent re-traumatization. Important elements of ensuring that FBMs are acceptable to and respectful of clients with trauma history could include 1) clinician awareness of the fact that clients’ experiences of trauma may shape their current functioning and response to treatment, 2) careful attention to creating a treatment atmosphere and clinical relationship in which clients are likely to feel safe and trusting and 3) promoting clients’ autonomy in treatment decisions (Champine et al., 2022; Grossman et al., 2021; Rajaraman et al., 2022).

Another closely associated mechanism might be the modulation of interoception-the signaling and perceiving of internal body sensations to maintain homeostasis (Farb et al., 2015; Craig et al., 2003). Interoceptive accuracy is decreased in chronic pain, (Di Lernia et al., 29 2020; Di Lernia et al., 2016; Blanchard et al., 1981; Duschek et al., 2017; Flor et al., 1992; 1999) and recent research links ACEs to reductions in self-reported interoceptive accuracy, which are associated with poorer mental health (Benavides and Brindle, 2025). Both gentle touch and deep pressure engage the insula, the central hub of the interoceptive network, (Craig et al., 2003; Craig and Craig, 2009; Craig, 2002; Pollatos et al., 2016; García-Cordero et al., 2017; Chen et al., 2021; Sammons et al., 2024) and deep touch and osteopathic manual therapy (Cerritelli et al., 21 2020) have been found to enhance interoceptive accuracy (Edwards et al., 2018). FBM practitioners often inquire about areas of perceived tension or restricted movement in the client’s body, guiding them to become aware of trauma or fear associated with physical symptoms in the body. This process is similar to somatic tracking, a component of PRT and other cognitive interventions that encourage interoceptive attention that and have successfully reduced chronic pain (Ashar et al., 2022; Mehling et al., 2024).

Another possibility, given these overlapping and potentially synergistic pathways, is the integration of soft tissue FBMs with cognitive-behavioral therapies, which we regard as an especially promising avenue. For example, manual therapists have shown interest in Pain Reprocessing Therapy (PRT), (Ashar et al., 2022; Pain, 2025) a recently developed intervention for nociplastic pain that teaches patients to view pain as a “false alarm” that emerges from “mind-body” processes rather than as an indication of bodily harm (Ashar et al., 2023). This shift in perspective is supported by inconsistencies in the timing and spatial distribution of pain that suggest an interoceptive contribution (Schubiner et al., 2024). Similarly, Pain Neuroscience Education-one component of PRT-is currently being incorporated into many pain treatments and has led to reductions in FM pain intensity (Suso-Martí et al., 2022) and symptoms of PTSD (Benedict et al., 2024). FBMs could be integrated to associate sensations (including pain) with safety and reduce avoidance behaviors. Again, trauma-specific, evidence-based training is essential to ensure efficacy and avoid harm (Cook et al., 2019).

### 2.6. Social effects

Another important way in which soft tissue FBMs may target the effects of trauma on pain is through social connection. Touch is fundamental for establishing social relationships, enhancing salience and promoting biobehavioral synchrony (Craig, 2002; Aureli and Presaghi, 2010; Fotopoulou and Tsakiris, 2017). Social touch can communicate emotion (Hertenstein, 2002; Hertenstein et al., 2006) and downregulate stress (Kidd et al., 2023). When a person signals distress (such as by crying), an empathetic observer will often respond with touch to help the person restore homeostasis (Shamay-Tsoory and Eisenberger, 2021). This cycle is mediated by neural circuits linking touch perception, shared distress, emotion regulation, reward, and synchronized brain activity in the observation-execution system (Shamay-Tsoory and Eisenberger, 2021). Social effects of touch are altered in individuals with PTSD, (Stevens et al., 2024) especially when the trauma involved touch.

A lack of early social connection and associated reductions in affective touch may impact long-term touch perception and social experience. In rat pups, early grooming levels influence oxytocin receptor levels (Francis et al., 2000; Champagne et al., 2001). In mice, touch deprivation leads to social isolation and a lack of social preferences, while CT-targeted stimulation increases the activity of oxytocin neurons and prosocial behaviors in adulthood (Yu et al., 2022; Huzard et al., 2022). In humans, individuals with emotional trauma history exhibit reduced blood plasma oxytocin levels, (Heim et al., 2009; Donadon et al., 2018b) potentially linked to differences in social touch experience. Effects of FBMs on oxytocin could be particularly valuable for patients with a fear of social touch, which can block intimate social connections their plethora of biopsychosocial benefits (Castonguay et al., 2010).

Reduced social touch also predicts loneliness, (Araújo et al., 2022) a prevalent symptom in both PTSD and chronic pain (Allen et al., 2020; Dagan and Yager, 2019; Fox et al., 2021; Loeffler and Steptoe, 2021). Two meta-analytic studies demonstrate that PTSD is strongly associated with reduced social support (Brewin et al., 2000; Ozer et al., 2003). Social disconnection is strongly linked to pain, (Karayannis et al., 2019; Eisenberger, 2012) suggesting a vicious cycle of reduced social touch, loneliness, social disconnection, and pain. In humans, some studies have observed that administering social touch reduces loneliness, but findings are mixed (Packheiser et al., 2025; Heatley Tejada et al., 2020). A recent review proposes that acute loneliness may enhance desire for social touch, while chronic loneliness may be associated with distrust and increased social threat perception, deterring social touch (Packheiser et al., 2025). Research is needed to test this hypothesis and whether carefully designed FBMs could help individuals who are low in social trust to increase their comfort with social touch; this would be expected to improve loneliness, social connection, and chronic pain.

More broadly, a trusting relationship between clinician and client, known as the therapeutic alliance, is a well-demonstrated building block of clinical improvement in PTSD (Howard et al., 2022) and other psychiatric conditions, with stronger therapeutic bonds associated with greater reductions in psychological distress (Horvath et al., 2011; Martin et al., 2000). FBMs may engage a similar therapeutic mechanism to that by which CBT remediates interpersonal dysregulation by providing a trust-based therapeutic social relationship in which relearning can occur. Indeed, interventions targeting social connection show promise for treating common psychiatric conditions (Cruwys et al., 2014; Taylor et al., 2020). Touch in a social therapeutic context can enhance the therapeutic alliance (Fotopoulou et al., 2022) and reduce feelings of social exclusion, (Fotopoulou et al., 2022) bridging the gap between client and therapist (McParlin et al., 2022). Therapeutic touch can also improve interpretation of sensory information (McParlin et al., 2022). Notably, clients often perceive FBM practitioners as knowing the client’s body better than they do, which may provide comfort and decrease feelings of isolation (Clark, 2019).

#### Summary

**Fig. 1.**
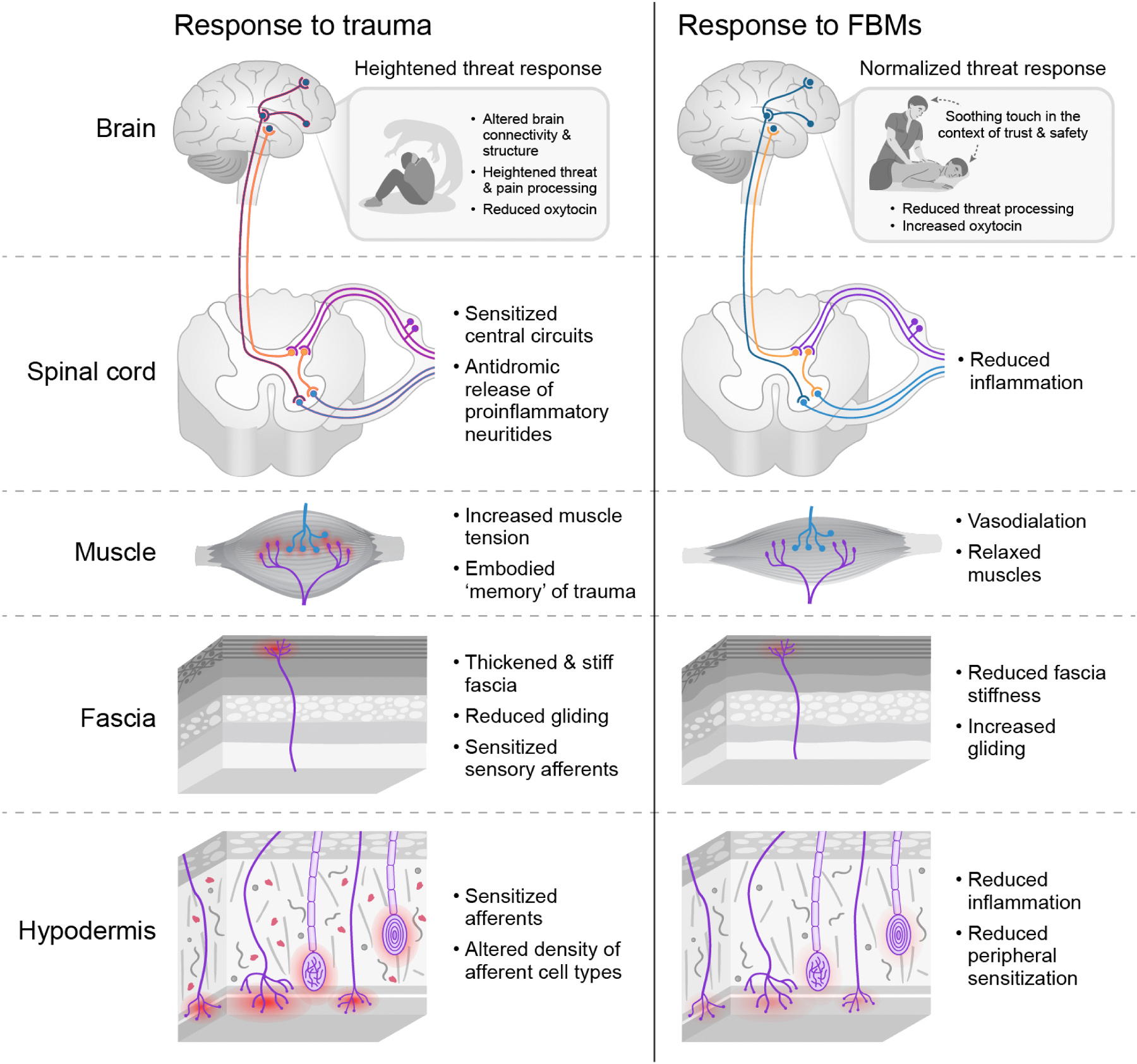
Summary of proposed effects of trauma on the somatosensory system and response to FBMs.

Trauma exerts far-reaching effects that sensitize both the peripheral and central components of the somatosensory and affective neurocircuitries towards nociception and threat-detection. The autonomic nervous system plays a key mediating role in most of these processes. See Fig. 1 and Table 1 for summaries of the effects of trauma and FBMs on the somatosensory system and soft tissues. By applying touch in a non-threatening context, soft tissue FBMs may act on multiple levels of the somatosensory system and on the fascial matrix in which the system is embedded to remediate these changes. Next, we consider this proposal through the lens of predictive coding, which considers the brain as a computational organ for anticipating the body’s future energy needs.

## 3. Towards an integrative ‘MASSAG’ theory of FBM mechanisms

Having considered the widespread impacts of stress and emotional trauma on different levels of the somatosensory and autonomic systems, we now propose an integrative theory for how soft tissue FBMs-when administered in a safe psychosocial context and ideally combined with psychological therapies-may make valuable contributions to the recalibration of these systems. We first introduce a predictive coding-based interpretation of changes in the systems with which FBMs interact and how those therapies might ameliorate post-traumatic conditions. We then propose the Mechanisms of Affective Somatosensory Soothing for Allostatic Gain (MASSAG), a model of mechanisms of soft tissue FBMs in the context of predictive coding, with massage therapy as an example. Finally, we identify research gaps and directions for further investigation of soft tissue FBMs that would fully consider the complex interplay between brain and body.

### 3.1. Predictive coding

Organisms must maintain their form in the face of a constantly changing environment and do so in an energetically efficient manner. To achieve this, they continuously sense and forecast their physiological needs, attempting to optimize autonomic, metabolic, and immunologic setpoints to face both current and expected metabolic demands – a process known as allostasis (Barrett, 2017). The better the forecast, the more ably the organism can anticipate demands without expending too much energy.

Predictive coding, a theoretical framework that considers organisms as fundamentally anticipatory, describes how the brain continuously predicts the external and internal milieus to regulate physiology and behavior with respect to emerging needs (Friston, 2010). According to this theory, the brain infers the hidden causes of sensory input by constructing probabilistic predictive models of future sensory input from the body and the environment based on past experience (Fig. 1). These predictions are generated continuously and are passed down the cortical hierarchy, while sensory data from the periphery is compared to descending predictions at each level. To evaluate and improve top-down predictions (or Bayesian priors) the brain computes the mismatch between predictions and bottom-up sensory input to obtain a prediction error. Prediction errors then travel up the hierarchy to ideally update higher-level priors and reduce future prediction errors. Moment to moment, the brain is thus inferring the most likely significance of sensory input by minimizing prediction errors across all levels, with the inference experienced as perception.

This process can be mathematically represented by the Bayesian weighing of evidence (sense data) and priors (predictions) to produce a posterior (perception). Ascending prediction errors and descending predictions are weighted by their relative precision or confidence, so that a high confidence prediction (or prediction error) carries more weight than a low confidence one in shaping the resulting percept. In other words, the brain not only predicts what sensory information will arrive but also how precise it will be, and it uses the latter to assign relative weights to predictions and prediction errors. More weight is given to prediction errors that result from predictions which were expected to be precise but turned out not to be, so that they update future predictions, and less weight is assigned to prediction errors resulting from predictions that were expected to be imprecise. Statistically, precision is the inverse of the signal’s variance, such that higher precision corresponds to a narrower probability distribution of sensory predictions and has a greater influence on the posterior distribution that is experienced as perception. For example, traversing a well-lit room (low-uncertainty condition), sensory input is highly precise, and the brain relies on its top-down predictions, but navigating a very dark room (high-uncertainty condition) makes sensory input imprecise, so the brain must either ignore the noisy sensory data or actively seek out more reliable data by altering behavior. Following the same logic, a brain that is convinced that the body is injured (high-confidence prediction) will assign low relative weight to ascending sensory data and would be more likely to experience pain even under weak or equivocal nociceptive input from the periphery. High-precision prediction errors, a signal that one’s internal model is off, therefore have privileged access to update higher-level beliefs, a configuration that minimizes future prediction errors while also minimizing the metabolically expensive process of updating beliefs (Kok et al., 2012; Brown et al., 2013; Veissière et al., 2020; Limanowski et al., 2020).

An organism can minimize prediction errors in multiple ways: It can update its models’ probabilistic priors to match lower-level sensations and priors, which amounts to calibrating perception; it can change sensory samples to align them with predictions by shifting attention, performing saccadic eye movements, or ignoring somatic sensation; or it can modify the environment through action so that it approximates the model’s priors (known as active inference). If models are updated, then prediction errors (or surprise) are minimized. The result is a predictive model that can predict the future states of dynamic internal and external environments without having to process the environments’ full sensory granularity moment by moment – a metabolically efficient solution. However, the brain must continuously calibrate its model of the body and the environmentto remain flexibly adapted to changes in both, with negative consequences arising when models are not plastic (capable of revision) enough (Seth and Friston, 2016).

### 3.2. Interoception and affect

Active inference in the context of the internal milieu is a special case of predictive coding, with afferent interoceptive signals playing a crucial role in the construction of both affect and pain (Seth and Friston, 2016). Predictive coding accounts of interoception argue that to perform allostasis, visceromotor cortices send predictions to the body to regulate autonomic and physiological setpoints. These cortical areas also send interoceptive predictions about their sensory consequences to the interoceptive cortex (mid-to-posterior insula), where ascending afferent sensory signals from the periphery (skin, muscle, connective tissues, and visceral organs) are used to compute prediction errors (predicted minus actual interoceptive signals), which are then propagated back to the visceromotor cortices (Barrett et al., 2016) (Barrett and Simmons, 2015). There, prediction errors are minimized, and the predicted interoceptive consequences of allostatic processes are experienced as interoceptive sensation (Craig et al., 2003). Put differently, descending interoceptive predictions establish homeostatic setpoints against which ascending interoceptive afferent signals are compared. Autonomic activation will track descending interoceptive predictions when prediction error is low, while larger prediction errors will drive corrective changes in sympathetic or parasympathetic activation and may update future predictions (see (Barrett and Simmons, 2015) for theoretical details).

**Fig. 2.**
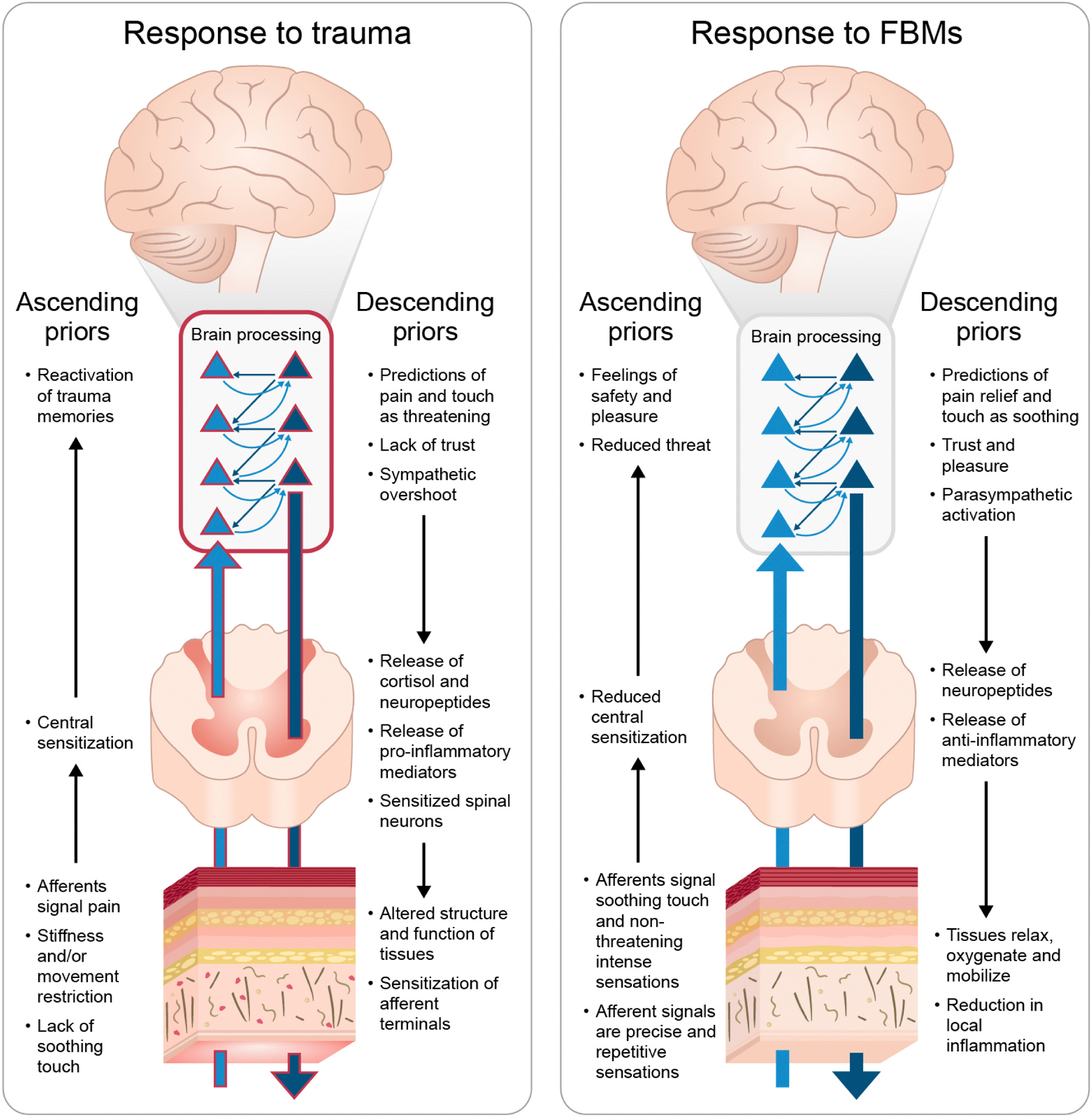
Visualization of the MASSAG model.

Pain-related prediction error signals have been observed in the ventral aINS, (Fazeli and Büchel, 2018) periaqueductal grey (PAG), (Roy et al., 2014) and the ACC-insula saliency network (Chen, 2023). These are modulated by anxiety and expectations (connectivity with ACC) as well as by positive expectations and inhibitions of threat responses (medial orbitofrontal cortex and hippocampus) (Tsai et al., 2024). The dorsal insula models interoceptive sensation based on ascending input from and descending predictions about somatosensory and visceral input from the skin, muscle, connective tissues, and visceral organs, and feeds interoceptive representations forward into subjective perception of our body, energy, and affective state (Fig. 2). Integration of interoceptive and exteroceptive (visual, vestibular, proprioceptive, and tactile) inputs then leads to the formation of higher-level body representations for that impact body ownership and agency (Kearney and Lanius, 2022; Longo et al., 2008; Seth, 2013; Gentsch et al., 2016; Tsakiris, 2017).

Feelings arise from the above-described prediction-driven interoceptive simulations of physiological needs (Craig et al., 2003; Barrett, 2017). Expectations about the causes of interoceptive signals are thus significant determinants of feelings (Barrett, 2017; Seth and Friston, 2016; Barrett and Bliss-Moreau, 2009). When a person unexpectedly steps on a nail, the nociceptive signal and consequent metabolic outlay generate a prediction error that will generate pain and negative affect. This prediction error may lead to minor corrections in behavior (walking more cautiously) but does not require revision of deep predictive models of the world; therefore, the negative affect is likely to be transitory. In contrast, if a formerly trusted partner acts with violence, a sympathetic response will generate fear and escape (metabolic outlay). In addition, this event will require the individual to significantly update their internal models of who and when to trust, requiring even greater and longer-term metabolic outlays. This will result in even greater and longer feelings of negative affect. Hence, affect results not only from interoceptive predictions about the immediate consequences of changes in the environment, but also from the accuracy of internal models themselves.

### 3.3. Memory, trauma, and stress

In the context of predictive coding, memory is not a passive storage system but rather an inference-driven process through which the brain actively reconstructs past experiences based on stored patterns. A memory can reactivate the interoceptive prediction that the original event evoked, triggering a reexperiencing of embodied sensations and feelings. Remembering a lover evokes the sensation of butterflies in the chest, and remembering a traumatic experience may evoke panic.

In predictive coding terms, an unexpected traumatic event is a salient threat that was not predicted and therefore triggers a large prediction error (Fig. 2), along with a sympathetic response that prepares the body for a large metabolic outlay to face the threat and the corresponding interoceptive sensations. The prediction error travels up the predictive hierarchy and remodels priors to increase the probability that the next time a similar context is encountered the threat will be correctly predicted. The remodeled priors reduce the probability of a similar future surprise at the cost of overpredicting similar threats. At the same time, long-term alterations of the peripheral systems reviewed above, i.e. neurological, immune, and fascial, sensitize those systems to message predictions of trauma to higher centers. Longer term, non-threatening events that evoke the threat’s original context now will carry a higher probability of triggering a threat prediction and sympathetic overshoot, increasing allostatic load. The reaction and associated sensations can be as salient and debilitating as the initial threat, potentially even developing into PTSD.

The threat predictions triggered by a predictive system marked by trauma or by an environment that is constantly perceived as a threat (i.e., a stressful one) cascade down to the periphery, where sympathetic flooding impacts the soft tissues, with both immediate and long-term consequences on neuroendocrine, neuromuscular, and peptidergic pathways. These include sympathetic-adrenal activation, neurotransmitter release from postganglionic neurons, intracellular signaling cascades (such as cAMP), contractility mediators like TGF-beta1, and neuropeptide signaling. In particular, neuropeptides are widely distributed signaling molecules released in response to emotional or traumatic events, (Morgan et al., 2002; Yang et al., 2018) which exert broad regulatory effects on multiple organs and tissues (Brain and Cox, 2006) and may participate in encoding “molecular memories” of traumatic experiences, (Van der Kolk, 2014) contributing to long-term alterations in muscle function and pain perception. Moreover, we hypothesize that sympathetic flooding by fascial autonomic effectors may provoke modifications of fascial components described in Section 2.3 that mimic those that attended the original trauma. The rapid and large outputs of epinephrine and norepinephrine from the sympathetic adrenal medulla triggered by sympathetic overshoots may produce a cascade of inflammation, densification, fibrosis, and nociceptive sensitization in the fascial matrix similar to effects of physical injury. Any long-term remodeling would become a peripheral component of trauma memory, with soft tissue becoming chronically inflamed, contracted, and hyperalgesic. Finally, new rounds of threat predictions, with corresponding sensory input from increasingly sensitized peripheral soft tissues, could create a positive feedback loop that “locks,” i.e. maintains the trauma in the tissue long term. Interoceptive somatosensory information that is compared to centrally generated predictions can itself contain information from past traumas, which when fed back up the line can bias the prediction model towards an expectation that similar trauma is about to occur. Traumatic memories may thus embed and embody themselves by acting directly on soft tissue in the periphery, accounting for why trauma often results in chronic autonomic dysregulation, muscle tension, fascial restriction, and altered cellular signaling that produce persistent pain and reduced mobility (Scaer, 2014).

As we will further explain below, we propose that FBMs could target tissue-level encoding of interoceptive and other components of traumatic memories and help to recode higher-level interoceptive predictions. From that point of view, they may be particularly effective at targeting peripheral components of embodied memory.

Beyond the periphery, traumatic memories and their associated revisions to allostatic prediction can modify the central nervous system in ways that further embed the traumatic memory and heighten its symptoms through the fear and attentional pathways described in Section 2.5. A person who has suffered through trauma will now constantly expect a threat when presented with cues related to the original context of the trauma. They may now view a stranger as likely to inflict harm or perceive friendly touch as aversive. In this way, distorted threat appraisals can become positive feedback loops.

Stress, anxiety, and trauma affect the strength of signaling at the most basic levels of interoception, as well as the ability to tolerate the disturbances, which in turn may compromise the accurate interpretation of sensations and related decisions regarding behavior (Price and Hooven, 2018). Chronic or severe stress affects interoceptive awareness by altering the intensity of internal cues as well as their perception and interpretation (Schulz and Vögele, 2015). Stress and anxiety disorders may be thought of in similar terms. When we are anxious, the brain interprets the environment or the future as ridden with danger, constantly predicting impending threats, while stress arises when the environment fails to match our predictions of it. In stress and anxiety disorders, the brain’s predictive models persistently fail to minimize errors in relation to threats or uncertainty, (Friston, 2010; Paulus and Stein, 2006) producing an ongoing mismatch between interoceptive predictions (e.g., expected heart rate, hormone levels) and incoming exteroceptive sensory signals, leading to sustained autonomic arousal and allostatic load (Sterling, 1988). Maladaptive priors and modifications of peripheral systems both exaggerate threat perception and perpetuate physiological and psychological dysregulation (Barrett et al., 2016).

### 3.4. Pain and pain relief

Like affect, pain appears to be actively constructed in the brain following a predictive coding logic. For example, the precision of pain predictions influences the strength of placebo hypoalgesia, (Büchel et al., 2014) and a recent study found that neural anticipation of pain is encoded in the anterior insula and in the nucleus accumbens and that this anticipatory coding differentiates individuals who consistently predict high pain, low pain, or neither under conditions of uncertainty (Strigo et al., 2022).

In predictive coding terms, chronic pain can be explained as an overestimation of prior beliefs in the context of a less noxious current stimulus, i.e., a ‘failure of inference’ (McParlin et al., 2022; Henningsen et al., 2018; Bohlen et al., 2021). Trauma and injury produce novel afferent signals that are initially outside of the predictive repertoire; as they are learned, priors are constructed to predict them, and nociceptive signals associated with them that may have been previously coded as innocuous now become noxious and painful. As already described, feedback between the predictive system and soft tissues can perpetuate local inflammation and hyperalgesia, which are more likely to generate ascending injury or threat signals. This can create a vicious cycle that keeps providing evidence for and strengthening the pain prediction.

Patients with chronic pain also tend to have maladaptive coping patterns of distracting *away* from bodily sensation, (Mehling et al., 2013) which, counterintuitively, increase pain (Goubert et al., 2004; Hasenbring and Verbunt, 2010). Since pain is aversive, a person suffering from persistent pain will tend to avoid stimuli that produce it. However, pain avoidance and escape prevent the predictive model from re-calibrating itself with sensory data to recognize that the nociceptive stimulus presents no real threat or from learning new pain offset expectations (expectations about when pain will end) congruent with tissue healing (Strigo and Simmons, 2024). This reinforces the existing encoding, perpetuating the pain. By preventing behavior that could recode pain priors, pain avoidance can maintain or worsen pain, trauma, and posttraumatic symptoms (Liedl and Knaevelsrud, 2008; Krause et al., 2008). Left untreated, an imprecise pain offset encoding can become linked to a sense of learned helplessness, a common complication among chronic pain patients (Linton and Shaw, 2011).

Conversely, therapeutic techniques that target the predictive architecture of pain (such as PRT, meditation, or exposure therapy) may be as or even more effective than pharmacological treatment. Indeed, having lower back pain sufferers reconceptualize the nature of their pain as due to nondangerous brain activity rather than peripheral tissue injury achieved greater pain reductions than standard of care or placebo (Ashar et al., 2022). Meditation techniques that involve sensorial body scanning alongside cultivation of a non-evaluative mindset reduce pain intensity and unpleasantness (Jinich-Diamant et al., 2020). In predictive coding terms these methods promote recalibrating pain priors to ascending somatosensory signals (Carhart-Harris and Friston, 2019). Interestingly, interoceptive training in patients with low back pain reduces experimental pain perception yet increases functional brain activation in the interoceptive network during pain (Strigo et al., 2024). This counterintuitive upregulation of pain processing despite pain reductions is consistent with greater metabolic needs for the recoding of predictive circuits and suggests that *interpretation* of interoceptive experience is key.

We postulate that by stimulating painful soft tissues with affective touch in a trust-based healing context, and in a repetitive manner calibrated so as not be excessively unpleasant, soft tissue FBMs can recode pain predicting priors, updating them with non-threatening sensory and affective signals. Soft tissue FBMs may also reduce chronic pain signaling by reducing densification and associated increase in fluid movement, increasing clearance of nociceptive amplifiers from interstitial fluid (Langevin, 2021; Kondrup et al., 2022; Pirri et al., 2025; Weiss and Kalichman, 2021). Both mechanisms would weaken self-fulfilling pain prediction loops and assuage pain conditions. Put differently, FBMs may facilitate pain relief by eliciting non-threatening and pain offset sensory signals in the moment and by helping to reduce nociceptive signals generated by inflamed peripheral soft tissue, especially in the context of a trusting clinician-patient relationship and coupled with other adjunctive affective, social, and cognitive therapeutic forms of top-down recalibration of pain-predicting priors. Of note, while affective touch may support reappraisal and regulation, it may also trigger trauma responses. Trauma-informed psychological therapies may be critical to accompany FBM sessions to more fully support processing of past trauma and reappraisal of emotional safety.

The clinical understanding of chronic pain conditions that involve mind-body connection and dysregulated interoception, like fibromyalgia (FM), can benefit from a predictive coding perspective and from considering FBMs as therapeutic interventions (Horsburgh et al., 2024). FM is a stress-evoked and sympathetically maintained neuropathic pain condition. The Fibromyalgia Imbalance of Threat and Soothing Systems (FITSS) model explains FM as driven by an overactive ‘threat’ system and an underactive ‘soothing’ system, an affective regulation imbalance which acts as a negative filter, keeping the salience network in continuous alert mode and contributing to central amplification of pain (Pinto et al., 2023a).

Individuals with FM have higher daily hassles and distress (Van Houdenhove et al., 2002) and a higher chance of early life adversity (Burke et al., 2017) than healthy peers, and physical abuse is the strongest predictor of developing FM (Kaleycheva et al., 2021). The resulting persistent threats and threat predictions, exacerbated by a lack of social support, produce a system that not only over-predicts threats but also under-predicts social validation and attachment, leading to a vicious cycle of negative affect, social disconnection, and underactive soothing and safeness. A stress-based overprediction of threats becomes anchored in self-fulfilling high-precision encodings that create a chronic state of sympathetic hyperactivity and allostatic load. Crucially, stressors may turn into neuropathic pain not only via higher-level mechanisms like the salience network, but also peripherally via alterations in dorsal root ganglia at the interface of the sympathetic and nociceptive systems (Martínez-Lavín, 2021) and alterations to fascial tissues (Langevin, 2021; Kondrup et al., 2022; Pirri et al., 2025; Weiss and Kalichman, 2021).

FM is of particular interest in this framework because it implicates all levels of the distributed somatosensory system as well as psychosocial, affective, peripheral, and central factors in a complex web of causation (Pinto et al., 2023b). The authors of the FITTS model suggest that FM treatments should aim to reduce threat perception and reinforce soothing abilities, but do not discuss touch or body-oriented therapies (Price and Hooven, 2018; Price et al., 2007). We propose that soft tissue FBMs act across the predictive hierarchy on each of the pillars of FITTS to reduce threat perception, reinforce soothing abilities, and change the brain’s negative filter into a positive one for trust and connection. For example, massage provides bottom-up sensory-afferent input via CT afferent stimulation and deep pressure, while also engaging top-down cognitive and affective processes by providing a psychosocial context conducive to trust and relaxation. Over time, repeated positive touch experiences and non-threatening activation of painful body areas amidst a relaxed and trusting environment and a healing-based relationship can strengthen emotional regulation and self-soothing, (McParlin et al., 2022) recoding sensory and affective predictive priors into ones that evoke less pain. Fascial manipulation may also contribute by restoring fluid movement and elasticity to soft tissues, upregulating the clearance of nociceptive amplifiers and cytokines which contribute to pain chronicity.

We propose that by targeting many of these factors simultaneously, soft tissue FBMs like massage therapy and fascial manipulation may offer exceptional therapeutic benefits for patients with FM and other mind-body pain conditions, particularly when used in combination with existing psychological therapies for trauma. While we focus on proposed benefits and mechanisms of FBMs because of their relative lack of research, our approach is intended to be inclusive and bidirectional, inviting broad integrative research combining FBM approaches with established therapies for trauma and pain. Indeed, body-oriented therapy, which includes massage and facilitated exercises to enhance focus and acceptance of internal sensory-emotional experience, has been shown to be a feasible and promising way to reduce physical and psychological distress in individuals with sexual trauma (Price et al., 2007; Price, 2006). Similarly, recent studies of ‘autonomic recalibration’ (AR) for chronic myofascial pain illustrate the effects that can be achieved by integration of an FBM with trauma-informed psychoeducation. AR uses palpation to assess startle response to normal touch combined with several manual and breathing techniques in a safe environment to target reflex loops and nociceptive trigger points. AR led to pain relief, improved sleep, and restoration of functional abilities in a diverse series of pain cases (Seton et al., 2024). In a subsequent pilot study, AR induced large reductions in pain ratings (*M* = 3.8 on NRS scale 0–11), muscle stiffness, and electrodermal response, while heart rate variability increased (Cawley et al., 2024).

### 3.5. The MASSAG model

We propose the Mechanisms of Affective Somatosensory Soothing for Allostatic Gain (MASSAG) model to describe the mechanisms of soft tissue FBMs, with massage therapy as our illustrative example. The MASSAG model proposes that FBMs provide high precision ascending sensory signals of soothing in a non-threatening context and that they modify soft tissues to reduce pro-inflammatory and nociceptive amplifiers, down-regulating embedded nociceptors. These signals ascend the predictive hierarchy and recode maladaptive and deep priors (or hyper-priors), recalibrating and diminishing threat prediction and conferring allostatic gain. See Fig. 2 for a visual depiction of the MASSAG Model.

Massage, for example, consists of a series of repetitive forces applied to the body at a wide range of velocities and pressures. Sensations of warming, cooling, pressure, vibration, and even discomfort are typically evoked, reflecting ascending sensory signals that are likely to be multimodal, novel, and both positively and negatively valent. Painful stimulation will contain a pain onset and offset, repeated multiple times, all without the previously associated injury or trauma-based threat and interspersed with non-painful stimulation to other areas. This will provide the system an opportunity to recode the pain-predicting priors associated with afferent traffic from that tissue with high-precision sensory signals and a new pain offset, which pain avoidance would have previously impeded. The salience of sensations is usually adjusted by the therapist and the patient to a level that “feels right”, i.e., one that may activate negatively valent sensations but not to the degree that they feel threatening. The salience and repetitive nature of the evoked, jointly calibratednon-threatening sensations increase the probability that they elicit a prediction error against descending pain or fear-associated predictions. This, in turn, facilitates the prediction error, now containing the new encoding, to ascend the predictive hierarchy and penetrate deeper inference layers to remodel future predictions with the new, more adaptive encoding offered by the evoked experience. This re-encoding can break pain avoidance loops and ingrained pain-predicting priors (see Fig. 2), especially when accompanied by cognitive, affective, and social changes that further support the prior remodeling process.

Critically, the MASSAG model involves affective touch as a safety signal. While previous models have discussed the importance of unlearning pain in a safe context and with biobehavioral synchrony of patient and provider, (McParlin et al., 2022) we additionally propose that specific forms of affective touch engage social brain circuity involved in detecting social proximity and safety while inhibiting threat perception. CT affective touch, as previously detailed, engages pathways associated with social connection, positive mood, oxytocin release, and parasympathetic regulation, (Van Puyvelde et al., 2019) while deep pressure and warmth can reduce sympathetic arousal, (Reynolds et al., 2015) increase parasympathetic activity, (Diego and Field, 2009) and decrease pain (Honigman et al., 2016). Increases in positive mood and social connection from affective touch in a caring environment facilitate decreases in the affective dimension of pain (which is modulated by mood (Villemure and Bushnell, 2002), 2009) as well as creating new positive associations for the environment in which pain and touch are experienced.

Attention towards and acceptance (non-avoidance) of bodily sensation is a key component of our model, increasing the salience and intensity of sensory signals that are used to recode predictions of threat and pain. This is facilitated by a restful, non-distracting environment of a typical FBM session where a patient lies with eyes closed and is guided to focus on the sensations provided or identified by the provider. This creates the conditions for high precision to be assigned to the ascending sensory signals (both pleasant and painful) produced by the therapeutic touch, which can be used to update the predictive model. This may be especially valuable for patients with trauma backgrounds, as FBM can reduce trauma-induced dissociative processes by reducing avoidance of bodily sensation (Price et al., 2007). Through a carefully choreographed multisensory combination of affective somatosensory input and interoceptive focus, self-fulfilling pain and fear-prediction loops become weakened, reducing the tissue and nervous-system embodiment of trauma.

The MASSAG model also identifies FBM-induced changes in peripheral tissues as a source of sensory afferent model updating. Massage and other soft tissue FBMs can increase tissue oxygenation, hydration, and mobility while reducing inflammation, stiffness, fibrosis, and excessive contractions, particularly in the fascia. These changes support improved tissue elasticity and reduced inflammation, directly targeting the chronic inflammation, contraction, and hyperalgesia produced by traumatic injury-induced densification, fibrosis, and sensitization. In addition to accelerating local tissue healing, soft tissue FBMs downregulate ascending nociceptive signals. Less inflamed tissues that generate less nociception are more likely to produce a prediction error with pain-expecting priors, thereby facilitating a new encoding of priors that predict less or no pain.

Soft tissue FBMs thus target a dysregulated interoceptive system that is constantly predicting threats (Miozzo et al., 2016; Kundakci et al., 2022; Bervoets et al., 2015; Furlan et al., 2015) simultaneously in a cognitive-affective (top-down) and sensory-afferent (bottom-up) manner, an approach particularly suited to the distributed effects of pain and emotional trauma. As self-fulfilling prediction loops are broken and interoceptive signals stop evoking threat memories that produce sympathetic overshoots, prediction errors become smaller and less frequent. Autonomic balance can improve and allostatic load decrease, resulting in more positive affect and greater openness towards learning, as greater precision can be placed on sensory data that no longer evokes embodied traumatic memories – a virtuous cycle of healing. Indeed, evidence shows that massage can often reduce feelings and biomarkers of stress, (Omena Bomfim, 2021; Noto et al., 2010; Lindgren et al., 2010) an index of allostatic load.

A novel feature of our model is the proposal that predictive processing is, to a significant degree, instantiated in the peripheral nervous system and the tissues in which it is embedded, not just in hierarchical organization of cortex. While pyramidal neurons in the cortex are often considered the primary cell type responsible for generating predictions and processing prediction errors (e.g (Klein et al., 2021; Allen and Tsakiri, 2019).), the tuning curve of a spinal projection neuron or a peripheral sensory afferent could also influence predictions. The sensitivity of afferent inputs and their weighting in the spinal cord bias predictions of the environment of the tissue and the level of threat it is expected to encounter, and increase the likelihood of corresponding sensory signals being sent to the brain. Peripheral afferents might instantiate predictions as their proportion of nociceptive end-organs increases after tissue injury, (Gangadharan et al., 2022) effectively hard-wiring a peripheral prediction for nociceptive input. Chronic inflammation can also sensitize sensory afferents in multiple tissue layers, and the spinal cord would respond to consequently upregulated nociceptive input, further amplifying nociceptive signaling and predictions of pain (Kondrup et al., 2022; Suarez-Rodriguez et al., 2022; Bradesi, 2010).

In the same manner, soft tissues may be also considered to instantiate prediction. We have extensively reviewed the tissue-level remodeling that can occur after trauma in muscle, fascia, and skin in the forms of tissue adhesion, thickness, restriction, inflammation, and reduced flow of interstitial fluids. A recent study even identifies a ‘skin memory’ for recent touch (Saal et al., 2023). When our fingertips touch a surface, forces are generated that change the viscosity and elasticity of the fingertip skin and alter the firing of mechanoreceptors. Some mechanoreceptors encode current force, while others encode recent force or a mix of both, and sensory afferents integrate this information to convey tactile information to the brain. An implication is that the force with which one is touched, as well as the force one applies to contact a person or object, will influence perception of future touch contact. This finding raises multiple questions for affective touch and FBM research and suggests that FBMs may reset ‘skin memory’ in ways that could alter perception of painful or pleasant touch. We hypothesize an exactly analogous mechanism in the mechanoreceptors which so richly innervate the deep fascia.

Just as pain and threat amplify one another by reinforcing pain-predictive priors, affective touch and a trust-based social context reinforce one another to diminish pain processing by creating new pain offset expectations, recoding pain priors as non-threatening, creating new affective touch sensory priors and new trust-based affective priors, altering local tissue states, and enhancing interoceptive signaling. Soft tissue FBMs thus operate across the distributed nature of the somatosensory system, targeting interoceptive input, affective state, predictive priors, and local tissue physiology. In contrast to the psychological and pharmacological approaches that make up today’s standards of care, soft tissue FBMs additionally provide safe exposure to both painful and pleasant touch, allowing for *immediate* changes in interpretation of sensory signals. These changes shift predictive models away from threat predictions, reducing sympathetic overshoot and allostatic load.

By updating interoceptive processes and relieving chronic nociceptive sensitivity, soft tissue FBMs also support an expansion of embodied agency, by fostering a multisensory re-integration of self-related signals across interoceptive and exteroceptive domains (Kearney and Lanius, 2022; Longo et al., 2008; Seth, 2013; Gentsch et al., 2016; Tsakiris, 2017). Resilient embodiment is fundamental for allostatic regulation as it supports the maintenance of physical integrity, (Seth and Tsakiris, 2018) functional competence, and distinction between one’s body and that of others (Cascio et al., 2012). Interoception has a unique role within this process, not only by providing information on the body’s internal state, which influences identity and agency, but also acting as a predictive framework against which exteroceptive information is mapped and interpreted, shaping our perception of the external world (Seth and Tsakiris, 2018; Tsakiris et al., 2011). Interoceptive inference and its role in multisensory integration controls physiological integrity, allostasis, and emotional regulation, (Seth and Tsakiris, 2018) and affords us a coherent sense of self and experience of the world and our place in it (Seth and Tsakiris, 2018). The role of FBMs in altering the phenomenology of embodiment would be a valuable research pursuit (Merleau-Ponty et al., 2013).

The MASSAG model synthesizes these mechanisms, proposing that soft tissue FBMs reduce threat perception and increase signaling of safety through multilevel, distributed changes in tissue state, sensory input, and predictive interpretation. By creating optimal physiological and psychological environments, FBMs enable clients to experience touch as a signal of safety rather than threat, and as relieving long-held distress within soft tissue physiology, gradually retraining the brain’s generative models to favor positive interpretations of bodily sensations. This framework aligns with exposure therapy principles, emphasizing the importance of repeated, safe touch experiences to downregulate threat responsivity associated with touch and physical sensations. We propose that, over time, these iterative processes reduce allostatic load, improve emotional regulation, and allow individuals to experience touch as a pathway to safety, connection, and healing. Note that some effects of FBMs may be delayed, as contextual factors and neuromodulatory changes (e.g., oxytocin responses, increased clearance of nociceptive and pro-inflammatory signaling) interact to shape future touch experiences (Handlin et al., 2023). Furthermore, there is currently insufficient evidence that FBMs can independently revise trauma-related priors; the co-involvement of psychological therapies engaging memory, cognition, or affective processing may be critical. This is an important topic for future research.

## 4. Summary and future directions

Emotional trauma triggers a cascade of responses through the brain and body that can lead to long-term alterations in multiple systems, which in turn alter the functioning of the somatosensory system. We argue that, at a mechanistic level, trauma reshapes the mind-body interface towards prediction of fear and pain, while soft tissue FBMs are ideally positioned to re-engage these targets to recalibrate prediction and interpretation, nudging the nervous system out of threat and pain perception and towards calm and restorative social connection. These methods may initiate sympathetic responses, tissue changes, and cycles of re-triggered interoceptive and proprioceptive signaling that mimic the original traumatic event. However, in a therapeutic context, this reactivation occurs alongside soothing sensory cues and a safe social relationship, allowing the brain to revise predictive models so that these signals may be reinterpreted as non-threatening. Over time, repeated safe exposure to intense and affective touch gradually updates these priors, and reductions in nociceptive sensitization in the soft tissues both reduce the brain’s bias towards pain- or trauma-based interpretations- and provide allostatic gain.

FBMs are understudied at the mechanistic level, both peripherally and centrally. To empirically test predictions of the MASSAG model, basic studies are needed to fill the many research gaps outlined above and to test in humans effects that have been observed in rodent models. Meta-analyses will be needed to estimate the strength of various effects and to compare the relative contribution of different mechanisms to the clinical effects of different FBMs. Then, clinical studies are needed to link both short and long-term FBM treatment outcomes to these validated mechanisms. Mechanistic research is needed to carefully disentangle the interrelated effects of stress, physical trauma, and emotional trauma, as well as persistent affective symptoms stemming from trauma (e.g., PTSD). One interesting question is the role of touch (or absence of touch) in mediating the effects of trauma. Given the activity-dependent changes in somatosensory afferents observed in early life in preclinical models (reviewed in Section 2.4), it is an urgent opportunity to study the effects of early touch input on somatosensory development in infants. Similarly, the plasticity of somatosensory innervation observed after injury, and changes in skin somatosensory cells after adolescent trauma, suggest that we are only beginning to understand how touch and pain can shape the somatosensory system. It will be important to study the developmental timecourse of such changes and whether FBMs might exert particular benefits during particular critical periods of development- or after injury or trauma. Touch interventions might additionally enhance the effects of pharmaceutical compounds like gabapentin, which can partially recover the appetitive value of pleasant touch in a rodent models of nerve injury (Zain et al., 2023).

Mechanisms of predictive coding are challenging to test because of the empirical difficulty of isolating predictive signals in the brain. However, recent studies have made headway. Individual differences in pain prediction have been identified the anterior insula and nucleus accumbens, (Strigo et al., 2022) and increased interoceptive processing of pain has been observed after interoceptive training (Strigo et al., 2024). Individual differences in brain activity in a frontoparietal network and decreases in a posterior insular/temporal network during pain anticipation are predictive of placebo analgesia, while decreased limbic and paralimbic activity predict placebo analgesia during pain (Wager et al., 2011). Furthermore, a recent study operationalized measurement of hierarchical cortical processing in pain by defining a minimal cortical pain network consisting of the lateral frontal pole, primary somatosensory cortex, and posterior insula. They then compared effective connectivity from resting state fMRI data between these regions in chronic pain versus healthy individuals and according to placebo response. Distinct patterns were observed of altered top-down, bottom-up, and recurrent (i.e., intrinsic) effective connectivity, allowing estimates of excitatory versus inhibitory forward connections (lower-to-higher cortical regions) and backward connections (higher-to-lower cortical regions). The observed effects were consistent with predictive processing accounts of placebo effects and chronic pain. We propose that similar approaches could be used to test changes in pain prediction before and after a series of soft tissue FBM (Nara et al., 2025).

At present, we propose that all studies of FBM mechanisms or treatment attempt to include measures of as many core MASSAG model components as possible, spanning multiple levels of analysis. Such designs are useful to fully understand each mechanism at an integrative level. Machine learning analysis of brain imaging studies will be needed to determine the effects of FBM treatments on neural pain predictions and pain responses, alongside behavioral and psychological measures of pain expectations, pain beliefs, and autonomic activation. Computational models of sensory uncertainty will be valuable in validating predictions of the MASSAG model. Graph neural network, transformer neural network, and multimodal neural network models may eventually be helpful to model predictive mechanisms and their interactions with the many components of FBMs.

Clinically, there is an urgent need to further study touch-based therapies for post-traumatic, anxiety-related disorders, given the underappreciated role of the skin and fascia as modulators of affective states and interoception (Müller-Oerlinghausen and Eggart, 2021). Somatic therapy has shown preliminary effectiveness for treating anxiety (Classen et al., 2021) and PTSD symptoms (Brom et al., 2017; Kuhfuß et al., 2021). Studies are needed to determine the impact of soft tissue FBMs on acute threat and pain response over time, and the role of brain networks such as the salience network in mediating threat-pain amplification. Research is also needed to determine how repeated FBM sessions, and the psychosocial factors associated with these sessions, may impact touch processing and pain modulation. Importantly, we suggest that approaches that utilize sensory-afferent input such as FBMs and exposure therapies may offer distinct additional benefits to those conferred by psychological therapies. We are not suggesting the replacement of psychological therapies, but rather propose to integrate FBM approaches to existing psychological treatment frameworks in future research and treatment efforts. We also suggest that many components of psychological treatments are already incorporated in some FMB treatments; these could be identified and enhanced through greater cross-disciplinary training efforts. We intend the MASSAG model to be an integrative scaffold where FBM adds value alongside other psychosocial, behavioral, autonomic, or educational interventions.

The MASSAG model is intended to guide both mechanistic and intervention research on how FBMs may benefit individuals with trauma history and chronic pain. We hope it inspires rigorous neuroscience, soft tissue, and clinical studies investigating underappreciated mechanisms the therapeutic benefit of FBMs.

## Figures and Tables

**Table 1:** Summary of proposed effects and evidence.

System	Effects of trauma	Evidence (selected)	Effects of FBMs	Evidence (selected)

ANS/HPA/Immune System	Sympathetic overshoot alters cortisol regulation	Seo et al. (2019) *N* = 73 adults; higher life trauma was associated with lower basal cortisol levels, mediated by increased acute stress responses in the amygdala and hippocampus Kuhlman et al. (2015) *N* = 97 adolescents; trauma during infancy delayed cortisol recovery from stress; trauma in later life altered circadian cortisol regulation	Parasympathetic activation; stress reduction; downregulation of HPA axis activity and cortisol levels	Moraska et al. (2010): review of human studies; 8 of 9 studies testing salivary cortisol observed a significant reduction after massage therapy Meier et al. (2020) *N* = 60 healthy women; massage significantly increased subjective relaxation and decreased subjective stress Diego and Field (2009) *N* = 20 healthy adults; moderate versus light pressure massage increased high frequency heart rate variability and decreased low/high frequency ratio Rapaport et al. 2010^83^: *N* = 53 healthy adults; Swedish massage versus light touch decreased arginine vasopressin and cortisol Cerritelli et al. (2020) *N* = 37 healthy adults; osteopathic manipulative treatment compared to sham showed parasympathetic effects on thermographic data, heart rate variability, and skin conductance
	Prolonged immune activation and persistent inflammation can lead to hyperalgesia and chronic pain	Iwai-Liao and Senba (2006) review of animal studies; repeated environmental stressors can induce a persistent hyperalgesic state Reichling and Levine (2009) review of numerous animal and human studies; supports casual effect of inflammation on acute hyperalgesia; ongoing stress elevates proinflammatory cytokines and increases their hyperalgesic effect	Lowers pain and inflammation	Crane et al. (2012) *N* = 11 young men; massage was associated with lower pain and inflammation following acute musculoskeletal pain from sports-related injuries White et al. (2020) *N* = 9 men; massage reduced inflammatory markers earlier than control after exercise
Muscle	Increases muscle contractility	(Lundberg et al., 1994) *N* = 62 women; stress tasks elevated EMG activity in trapezius muscle	Relaxes muscles and reduces muscle contractility	Bakar et al. (2014) *N* = 45 women; connective tissue massage increased muscle relaxation measured by EMG Eriksson Crommert et al. (2015) *N* = 18 healthy adults; massage decreased muscle shear elasticity immediately following massage (temporary)
	Increases in muscle thickness and restriction	*Research gap*	Increases collagen in tendons; decrease adhesions, decrease stiffness	Kassolik et al. (2013) *N* = 18 rats; massage increased collagen fibrils in tendons Bove and Chapelle (2012) *N* = 30 rats; visceral mobilization decreased number of visceral adhesions and adhesion severity after abdominal surgery Bove et al. (2017) *N* = 147 rats; manual therapy after surgery reduced frequency and size of cohesive adhesions Devantéry et al. (2023) *N* = 49 adults with chronic non-specific lower back pain; myofascial technique reduced muscle stiffness
	Reduces local perfusion and oxygenation	*Research gap; indirect evidence* D’Souza et al. (2024) *N* = 31 women with PTSD demonstrated greater muscle fatigability during exercise and blunted peripheral extraction of oxygen Puel et al. (2023) *N* = 53 adolescents with temporomandibular disorder exhibited elevated stress and reduced masseter oxyhemoglobin values	Increases skin perfusion and muscle oxygenation	Monteiro Rodrigues et al. (2020) *N* = 32 healthy adults; massage increased local perfusion Soares et al. (2020) *N* = 12 healthy men; forearm muscle oxygenation transiently increased after brief rolling massage
Fascia	Pro-inflammatory cytokines, tissue densification, increases collagenous inelastic fibers, decreases tissue glide, decreases lymphatic drainage, and increases nociceptive innervation	Kondrup et al. (2022) review of human studies of deep fascia pathology and pain; identified increased tissue stiffness, alterations in myofibroblast activity and the extra-cellular matrix, and increased density and sensitization of nociceptive nerve fibers, and pro-inflammatory cytokines and immune cells Barbe et al. (2013) *N* = 275 female rats; repetitive motion injury led to chronic inflammation with macrophage infiltration and fibrosis of deep fasciae and perineural tissues Langevin et al. (2011) *N* = 121 adults with chronic low back pain; thoracolumbar fascia shear strain reduced in patients with chronic low back pain Vita et al. (2025) *N* = 79 adults with chronic low back pain; significant correlation between muscle hypotrophy, thickness of the thoracolumbar fascia and duration of onset of symptoms Tamartash et al. (2023) *N* = 68 adults with low back pain and *N* = 63 adults without low back pain; reduced elasticity of the thoracolumbar fascia in patients with low back pain *Research gap-effects from stress and trauma*	Normalizes fascial thickness, stiffness and glide Reduces soft tissue pain	Overmann et al. (2024) *N* = 128 adults with chronic neck pain and depression; myofascial release reduced stiffness and increased range of motion of the cervical spine Devantery et al. (2023) *N* = 49 adults with chronic non-specific lower back pain; myofascial technique reduced fascial thickness Tozzi et al. (2011) *N* = 120 adults with neck or back pain; myofascial release improved pain ratings and ultrasound measures of sliding fascial mobility Pawlukiewicz et al. (2022) *N* = 54 young adults with MSK pain; fascial manipulation produced significant pain relief Ajimsha et al. (2015) review of human studies; myofascial release is effective for reducing pain (low back pain, neck pain and headache), and improving functional outcomes
	Central sensitization can cause downstream dysfunction in myofascial unit	Sikdar et al. (2023) proposed model based on clinical observations and several segmental effects of inflammation and autonomic response in rodents and humans *Research gap-effects from stress and trauma*	Reduces central sensitization may reverse dysfunction in myofascial unit	*Research gap*
Skin and associated Sensory Afferents	Stress, pain, or touch deprivation can alter development and sensitivity of somatosensory afferents	Santiago et al. (2023) mice; deletion of mechanosensitive ion channels altered end organ structure and central targeting of somatosensory afferents, especially CT fibers Harbour et al. (2025) *N* = 8–35 per group of neonatal mouse pups; early life stress altered transcriptional and electrophysiological signatures of dorsal root ganglia cells and led to touch and pain hypersensitivity Gangadharan et al. (2022) 3–9 mice per group; nerve injury led to ingrowth of nociceptive afferents and reduced low-threshold touch afferents Morrison et al., (2022) *N* = 101 women with sexual trauma; adolescent sexual trauma was associated with unique keratin-related proteins from EVs *Research gap-long-terms effects of early touch (or lack of touch) and pain*	Increases positive affect and reduces pain	Meijer et al. (2022) reviews neurophysiology of CT afferent system and its ability to reduce pain Loken et al. (2009) *N* = 16 healthy adults; mean firing rate of CT fibers correlated with pleasantness Pawling et al. (2017) *N* = 34 healthy adults; CT touch increased approachability of neutral faces and was associated with greater heart rate deacceleration and lower skin conductance responses Liljencrantz et al. (2017) *N* = 44 healthy adults; CT-optimal slow brushing reduced experimental heat pain Di Lernia et al. (2020) *N* = 49 adults with chronic pain; interoceptive stimulation of circular CT-activating brushing reduced perceived pain in chronic pain patients *Research gap-long-term effects on somatosensory afferents*
Brain and Cognitive Effects	Alters brain-wide connectivity and structure, especially brain regions involved in executive function and stress response	Kaul et al. (2021) review of macro/micro-structural data from animals and humans; provides support for stress-induced alterations of brain structure and connectivity Wang et al. (2019) *N* = 217 healthy adolescents; higher stress levels were associated with altered resting state connectivity	Normalizes brain connectivity and structure	*Research gap*
	Increases pain and threat response to touch; reduces touch pleasantness	Defrin et al. (2008) *N* = 32 adults with PTSD, *N* = 29 with anxiety disorder, and *N* = 20 healthy controls; PTSD was associated with higher rates of chronic pain, more painful body regions, and PTSD severity correlated with chronic pain severity Devine et al. (2020) *N* = 19 adults who spent time in foster care and *N* = 32 who did not; fostered adults reported higher levels of childhood trauma and showed reductions in sensitivity to affective value of CT targeted touch	Updates pain beliefs and reduces fear; increases touch pleasantness	Price (2006) *N* = 8 women with sexual trauma; body-oriented therapy reduced physical and psychological distress Cawley et al. (2024) *N* = 20 patients with chronic myofascial pain; manual and breathing techniques induced large reductions in pain ratings, electrodermal response, and nociceptive startle response; heart rate variability increased
Social effects	Reduces blood oxytocin levels	Heim et al. (2009) *N* = 22 women with childhood abuse or neglect; exposure to maltreatment was associated with decreased cerebral spinal fluid oxytocin concentrations Donadon et al. (2018b) review of 35 human studies; supports effects of emotional trauma and PTSD on reduced endogenous oxytocin	Gentle touch increases activity of oxytocin neurons and prosocial behaviors	Huzard et al. (2022) 20 mice; transient increases in C low-threshold mechanoreceptors excitability increased contact between animals, decreased isolated behavior, and had prosocial influences on group dynamics Yu et al. (2022) 2–7 mice per condition; social touch like stimulation increased activity of hypothalamic oxytocin neurons, induced conditioned place preference, and increased social interactions
	Increases loneliness, reduces social connection	Fox et al. (2021) *N* = 1276 aging adults; PTSD and loneliness were associated over time Brewin et al. (2000) & Ozer et al. (2003) Two meta-analytic studies demonstrating PTSD is strongly associated with reduced social support, for both prospective and retrospective study designs	Improves trust and social connection	Howard et al. (2022) review of 34 human studies supports the importance of therapeutic alliance for clinical improvement in PTSD Taylor et al. (2020) *N* = 29 adults seeking treatment for depression or anxiety; amplification of positivity protocol increased social connectedness Cruwys et al. (2014) *N* = 52 adults at risk of depression and *N* = 92 adults diagnosed with depression; social identification predicted recovery from depression in both groups
